# Implementing OECD GLP principles for the evaluation of novel vector control tools: a case study with two novel LLINs, SafeNet^®^ and SafeNet NF^®^

**DOI:** 10.1186/s12936-022-04208-4

**Published:** 2022-06-11

**Authors:** Salum Azizi, Janneke Snetselaar, Robert Kaaya, Johnson Matowo, Hudson Onen, Magreth Shayo, Ezekia Kisengwa, Evod Tilya, Baltazari Manunda, Benson Mawa, Franklin Mosha, Matthew Kirby

**Affiliations:** 1 Department of Medical Parasitology and Entomology, Kilimanjaro Christian Medical University College (KCMUCo), Moshi, 255 Tanzania; 2 Pan African Malaria Vector Research Consortium (PAMVERC), Moshi, 255 Tanzania; 3 Innovative Vector Control Consortium (IVCC), Liverpool, L3 5QA UK; 4 London School of Hygiene and Tropical Medicine (LSHTM), London, WC1E 7HT UK; 5 Department of Zoology, Entomology and Fisheries Sciences, College of Natural Sciences, School of Biosciences, Makerere University, P.O. Box 7062, Kampala, Uganda

**Keywords:** Good laboratory practice, SafeNet NF^®^, SafeNet^®^ LLIN, Interceptor^®^ LLIN, Experimental hut, *Anopheles arabiensis*

## Abstract

**Background:**

To sustain high universal Long-Lasting Insecticidal Nets (LLINs) coverage, affordable nets that provide equivalent or better protection than standard LLINs, are required. Test facilities evaluating new LLINs require compliance to Good Laboratory Practice (GLP) standards to ensure the quality and integrity of test data. Following GLP principles allows for the reconstruction of activities during the conduct of a study and minimizes duplication of efficacy testing. This case study evaluated the efficacy of two LLINs: SafeNet NF^®^ and SafeNet^®^ LLIN.

**Methods:**

The study was conducted according to GLP principles and followed World Health Organization guidelines for evaluating LLINs. The LLINs were assessed in experimental huts against wild, pyrethroid-resistant *Anopheles arabiensis* mosquitoes. Nets were either unwashed or washed 20 times and artificially holed to simulate a used torn net. Blood-feeding inhibition and mortality were compared with a positive control (Interceptor^®^ LLIN) and an untreated net.

**Results:**

Mosquito entry in the huts was reduced compared to negative control for the unwashed SafeNet NF, washed Safenet LLIN and the positive control arms. Similar exiting rates were found for all the treatment arms. Significant blood-feeding inhibition was only found for the positive control, both when washed and unwashed. All insecticide treatments induced significantly higher mortality compared to an untreated net. Compared to the positive control, the washed and unwashed SafeNet NF^®^ resulted in similar mortality. For the SafeNet^®^ LLINs the unwashed net had an equivalent performance, but the mortality for the washed net was significantly lower than the positive control.

Internal audits of the study confirmed that all critical phases complied with Standard Operating Procedures (SOPs) and the study plan. The external audit confirmed that the study complied with GLP standards.

**Conclusions:**

SafeNet NF^®^ and SafeNet^®^ LLIN offered equivalent protection to the positive control (Interceptor^®^ LLIN). However, further research is needed to investigate the durability, acceptability, and residual efficacy of these nets in the community. This study demonstrated that GLP-compliant evaluation of LLINs can be successfully conducted by African research institutions.

**Supplementary Information:**

The online version contains supplementary material available at 10.1186/s12936-022-04208-4.

## Background

Long-lasting insecticidal nets (LLINs), are among the main tools for malaria vector control. The World Health Organization (WHO) recommends universal coverage of LLINs [1] for all populations at risk of malaria transmission. LLINs averted 68% of malaria cases between 2000 and 2015 [2]. Globally, LLINs campaigns delivered approximately 250 million nets in 2019 [3]. Despite this effort, global LLINs coverage remains inadequate, with 56% of the population in endemic areas estimated not having access to an LLIN [3]. Across the African continent, there is a variable pattern of LLIN coverage, caused by several reasons including financial shortages, the logistics involved with re-supply, and net misuse and damage [4]. Notably, the global investment for malaria has remained relatively constant in the past 10 years [3]. The World Malaria Report of 2021 [3] stated that the allocated funding for malaria control and elimination was estimated at US$ 3.3 billion against a target of US$ 6.8 billion, i.e. less than half of the funds required to meet the WHO target to reduce malaria incidence and mortality by 40% by the year 2020. The 2021 version of the Global technical strategy for malaria 2016–2030 sets the target of reducing global malaria incidence and mortality rates by at least 90% by 2030, thus more investments needed. This has raised concerns about the future of LLIN coverage, and a renewed interest in looking for additional private sector investment to finance, produce and deliver affordable LLINs. There is a worrying trend of gains in malaria control stalling, resulting in less than 50% of endemic countries currently on track to reach critical malaria reduction targets [3].

From 1960, the coordination of assessing products for public health has lain with a division of the WHO, the WHO Pesticide Evaluation Scheme, which evaluated vector control tools such as LLINs and indoor residual sprays [5]. In recent years the process of evaluating new vector control products by the WHO has undergone a transition process from the WHOPES to the more recently established Prequalification Unit Vector Control Product Assessment Team (PQT/VCP) [6]. As part of this transition, the product testing has shifted from WHO collaborating testing institutions to GLP compliant sites. Compliance to GLP principles allows (1) mutual acceptance of data (2) prevention of duplicating testing and (3) ensures reliable, auditable, and reproducible data [7]. By focusing on the generation of data under a quality management system, PQT strives to enhance transparency in the product evaluation process. This development requires a shift of vector control research facilities towards GLP compliance [8], including African institutions, for which the process of achieving and maintaining GLP compliance may be challenging [9].

The importance of quality assurance in efficacy testing and manufacturing standards was showcased by the recent Global Fund report on substandard LLINs, describing the purchase of 52 million faulty LLINs worth $106 million between 2017 and 2019 [10]. To avoid this, WHO PQT-VCP was mandated to cooperate with national regulatory agencies and partner organizations, to ensure the efficacy and quality of vector control products [8, 11]. As a consequence, the WHO PQT-VCP has assessed and qualified 21 LLIN brands [12], which are acceptable for procurement by the UN and other international agencies or countries. Furthermore, the WHO PQT-VC emphasizes the need for test facilities involved in the evaluation of mosquito control products to comply with GLP standards [8].

The objective of this study was to showcase how GLP standards were implemented in an African trial institute for the evaluation of two novel LLINs; SafeNet NF^®^ and SafeNet^®^ LLIN. Both products were tested in laboratory and experimental hut trials at the Kilimanjaro Christian Medical University College (KCMUCo)- Pan African Malaria Vector Research Consortium (PAMVERC) Test Facility in Moshi, northern-Tanzania. The study reports were intended to be used for assessment of the two insecticidal products by WHO PQT-VC.

## Methods

### The GLP procedures

**Fig. 1:**
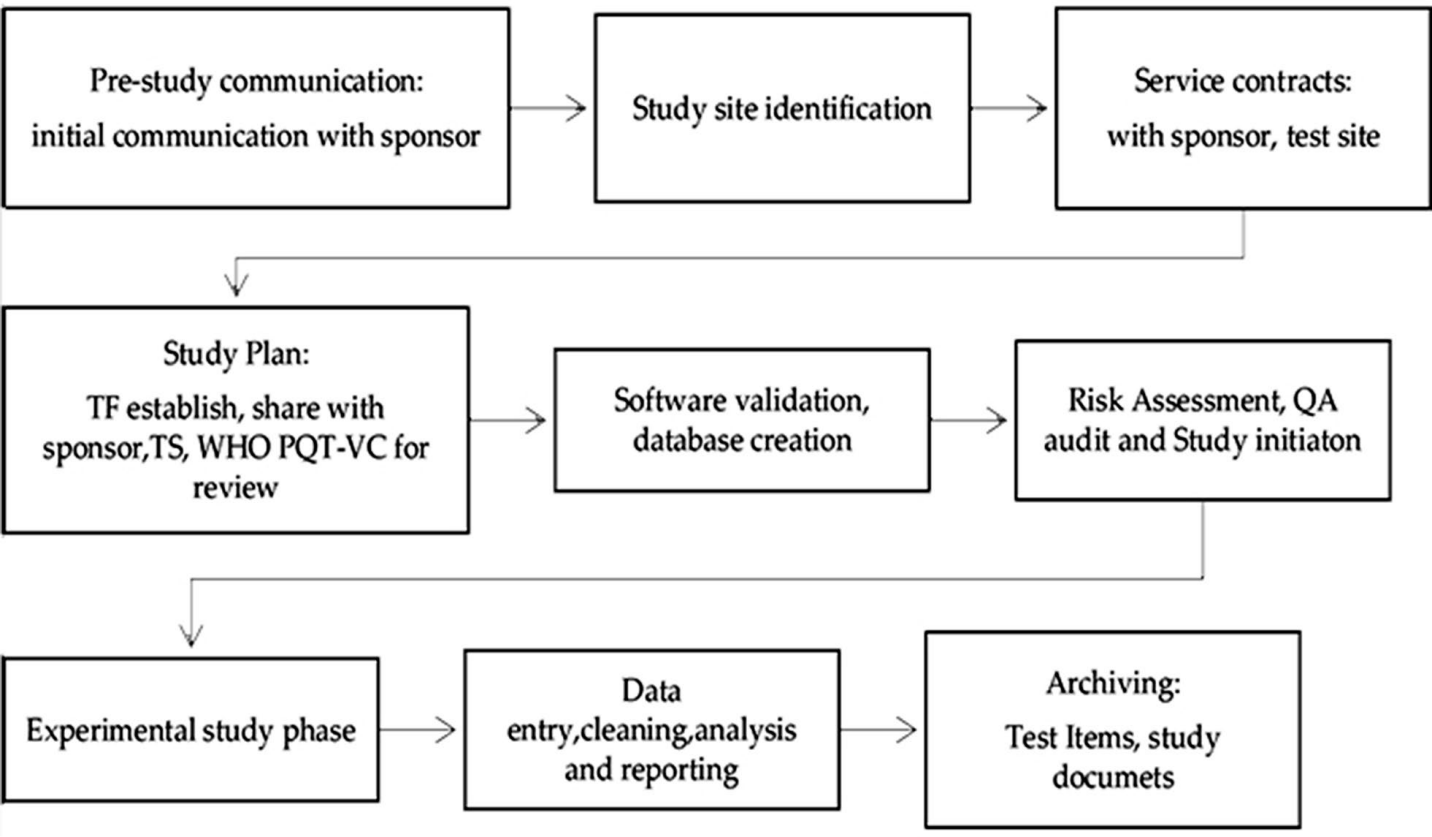
Map indicating the Pasua field station in Moshi, Northern-Eastern Tanzania, where the field evaluation of the LLINS was conducted

This study adhered to the following procedures to ensure GLP compliance, summarized in the study flow (Fig. 1).

#### Pre-study meetings

Before study initiation, a series of meetings with the sponsor were held to agree on the contract, timelines, and study objectives. Standard Operating Procedures (SOPs), safety data and certificate of analysis, WHOPES guidelines and GLP standards were shared. Development of the study plan, including the submission to WHO PQT-VC for review, was agreed. Parallel to this, a meeting was held at the Test Facility by the Test Facility Manager (TFM) to appoint a responsible Study Director (SD) for this study. The Test Facility Management ensured that the facility had sufficient space, infrastructure, manpower, and materials to conduct the study.

#### Identifying subcontractor for HPLC analysis

The Test Facility (TF) identified and shared details of two GLP compliant test sites for conducting High-Performance Liquid Chromatography (HPLC) for chemical analysis. One test site, Bio Genius GmbH (Germany) was chosen for doing chemical analysis, based on the availability of Test Site (TS), GLP certification, and costs.

#### Study plan and Implementation plan

The standard format for the study plan [13, 14] was used with consideration to the OECD GLP standards [7]. The study plan was shared with the WHO PQT-VC for review and recommendations. An implementation plan was established to describe the order of events in the study. Both implementation and study plan were prepared by the TF and shared with the sponsor and TS for review and agreement prior to implementation.

#### Study opening and the master schedule

The study was allocated with a unique code and added to the master schedule.

**Fig. 2:**
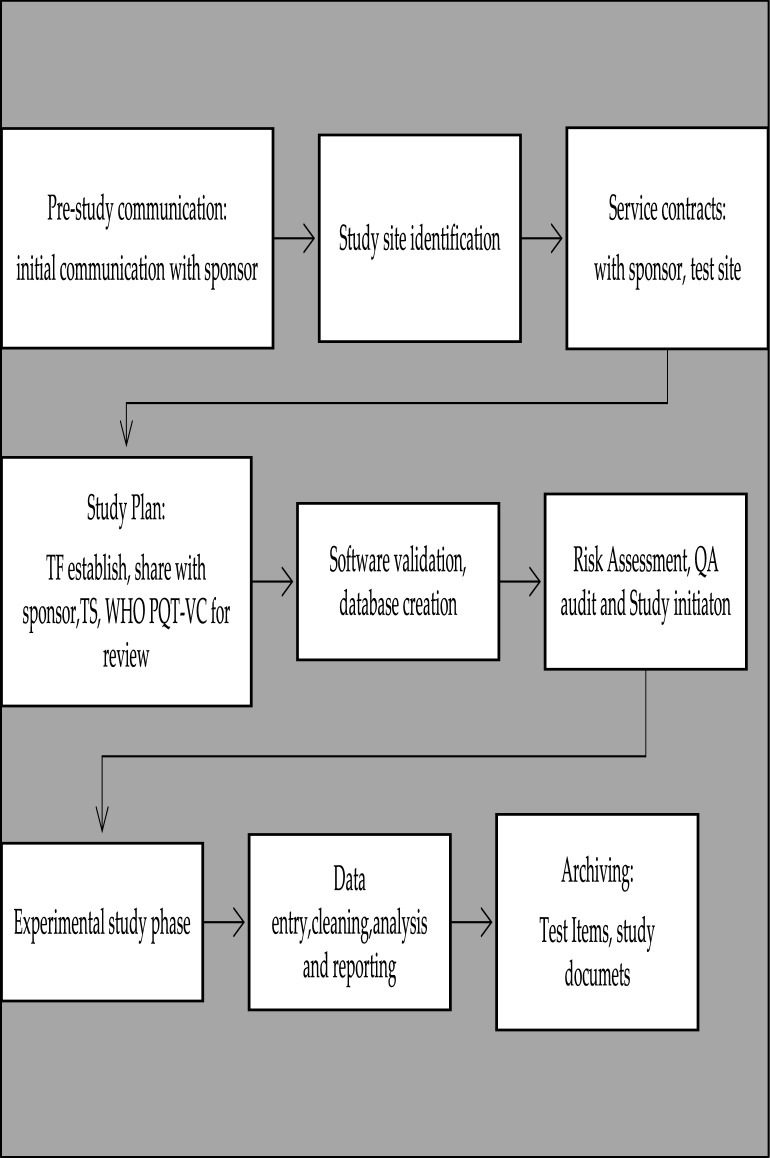
GLP study work flow. The boxes indicates the GLP procedures performed from the study initiation to the study completion

During monthly master schedule meetings, the SD and TFM recorded the dates on which the study passed through a series of study phases (Fig. 2). Additionally, the number and type of data forms were communicated to the data manager to establish a necessary database for data entry.

#### Study initiation and experimental phases

Once all review comments were incorporated in the study plan, the study plan was signed by the SD, TFM, Test site Manager, Test Facility Quality Assurance (QA) Manager, and the Test Site QA Manager. A staff-briefing meeting was conducted at the TF, where the Study Director explained the study plan in detail including the test items, test systems, the overview of the relationship between the Test Facility (KCMUCo-PAMVERC) and Test Site (Bio Genius, Germany).

#### Study risk assessment and mitigation

The study plan was submitted to the Health and Safety officer to identify any potential risk associated with conducting the study and to recommend measures to minimize health risks. In Tanzania, the risk assessment is a requirement by law [15]. Study activities that were assessed are; net cutting, washing, cone bioassays, bottle bioassays and mosquito collection during the hut trial.

#### Quality assurance auditing for study plan and identification of critical phases

To ensure that the planned study adhered to GLP principles the QA Manager from the TF and the QA Manager from the TS conducted an audit of the study plan. Subsequently, the QA Manager, Project Manager, and SD discussed the SOPs and study plan, and identified appropriate critical phases for QA audits. The critical phases are key activities, where deviation from SOPs and study plan/protocol could lead to misinterpretation of study outcomes.

The following critical phases were identified for auditing: net washing at the time of the 10th wash; conducting cone bioassays; rotation of treatments, collection of mosquitoes, and scoring of primary outcome measures.

The critical phases for the study activities that were conducted at the TS were identified by Test Site QA and Principal Investigator and were included in the overall audit report by the QA manager from the TF. At the TS, the experimental determination of alpha-cypermethrin content in long-lasting insecticidal nets was audited.

#### Critical phase auditing

The QA manager attended the 10th washing of the whole nets, and assessed the washing procedures together with the records of all previous washing. The QA also assessed conducting cone bioassays; rotation of treatments, collection of mosquitoes, and scoring of primary outcome measures. The Critical phase audits was conducted with reference to the Study Plan, relevant SOPs, and GLP principles. The Critical Phase audit report was discussed between SD, TFM and QA. Equivalently, the QA manager at the TS assessed the experimental determination of alpha-cypermethrin content the test items.

Using the critical phase checklist, the QA manager also inspected training and competence record of staff involved in a study, availability of SOPs and the study plan at the study area, maintenance records of equipment, test item, and test system record sheets.

#### Data management, software validation, and accuracy checks

Raw data collection, the data entry procedure, databases preparation, computers and software validation, comparison and accuracy check plans for the study were reviewed and discussed between the SD and the data manager. Custom TF’s developed validation checklists and validation kits for computer systems that ensure data integrity were followed by the data manager. These checklists are used to review whether logging, relocation, backup, and maintenance of all computerized system equipment, intended for use in this study complied with the OECD-GLP standards. Software that was validated included Mintab 17, MS Excel and Stata (Stata Corp LLC). The data exchange process from LIMS software AriaMx to MS excel Office was also validated. The Study Director reviewed the validation and approved the computer systems for the study.

#### Record of procedures

In this GLP study, the record of procedures, which records specific actions that involve the test items in the experimental procedures, was used throughout the study. Recorded experimental procedures included: cutting net pieces, washing nets, deliberately holing of nets, collection of test systems during hut trial, scoring of immediate and delayed mortality, packing of test systems, cutting of net pieces for bioassays, the conduct of bioassays, scoring of bioassay outcome measures, shipment of test items and archiving of test items. In the record of procedures information on equipment used, test items, test systems, technician initials, and date were recorded.

#### External quality assurance audit

The SANAS conducted interviews with TFM, SD, QA Manager, Data Manager, Archivist and some study personnel. Furthermore, SANAS employed the Checklist for OECD GLP No.1 (F-42) to assess study and facility compliance to GLP standards.

#### Final report

The final report, which is an ordered formal statement of the certification, quality assurance, study personnel, objectives, methods used, analysis, results and conclusions of a study was written and signed by the Study Director. This report was then counter signed by the lead QA Manager, TFM, and the Sponsor of the Project.

#### Archiving

At the end of the study, the original study folder, containing; the study plan, schedule of activities, correspondence with sponsors, amendments and deviations, records of procedures, the raw data, ancillary study data, analysis print-outs, and final reports, was archived at the test facility for five years. Also, one new unwashed unused net piece of each treatment was retained in the Test Facility and archived for 1 year. All used or expired test items were disposed of with the sponsor’s consent after the study. The dried carcasses of the test systems analysed in the molecular laboratory were being archived for 5 years following internal SOPs.

#### Waste disposal

All waste generated in the course of the study was disposed of in accordance with the OECD GLP document number 19 [7], the Test Facility waste disposal SOPs and recommendations from the National Environmental Management Council of Tanzania (NEMC) as detailed elsewhere [16].

#### Experimental hut site

**Fig. 3:**
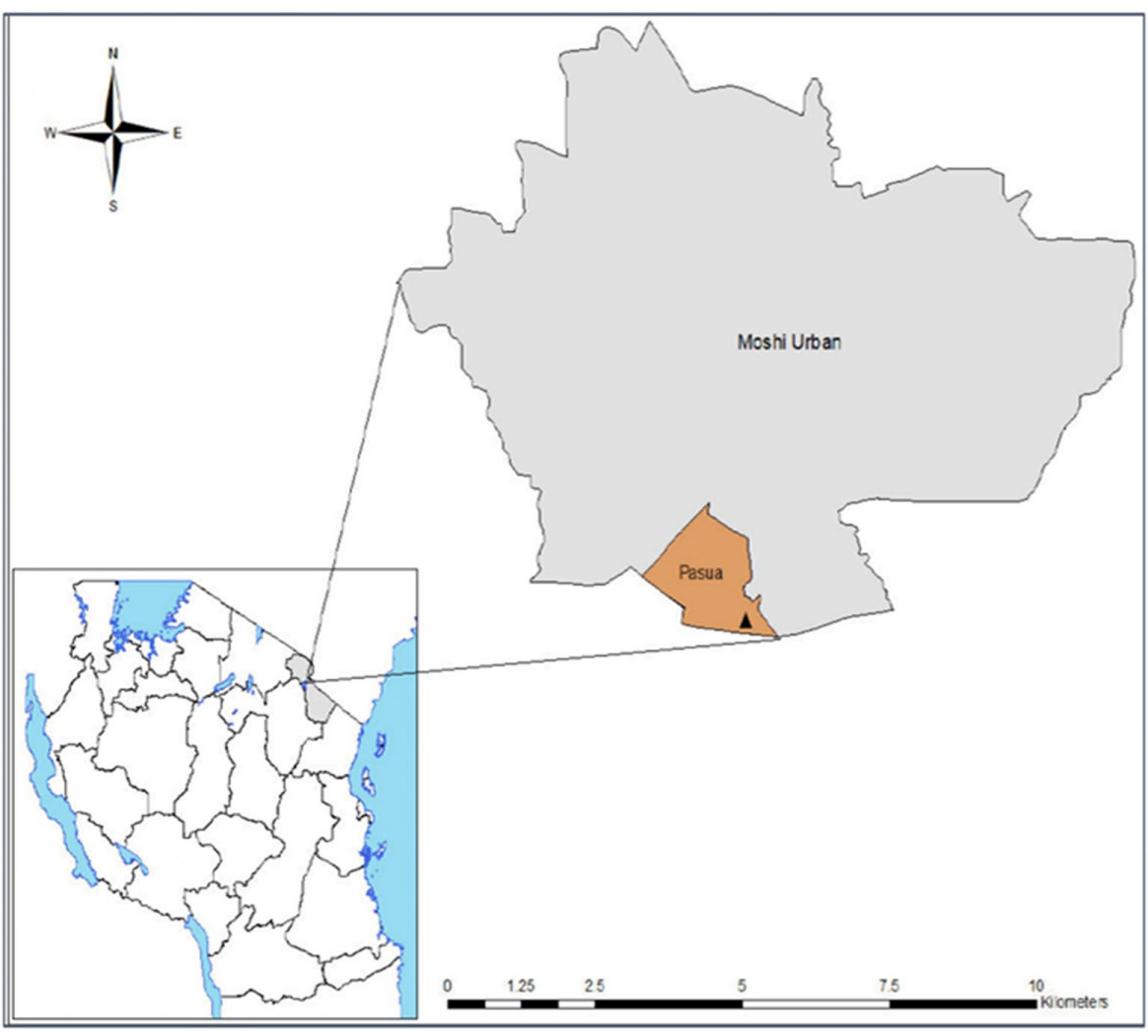
Phases for GLP study; from pre-planing to archiving phase

The field evaluation of the LLINs was conducted in the East African style experimental huts, located at Pasua (S03˚22.764′ E037˚20.793′), in the Lower Moshi adjacent to Lower Moshi rice irrigation scheme (Fig. 3). The irrigation scheme is getting water from the Rau river catchment, providing a reliable breeding site for local malaria vectors, *Anopheles arabiensis*. In this area, *An. arabiensis* has moderate pyrethroid resistance due to elevated levels of both mixed-function oxidases and β-esterases [17, 18].

### Experimental evaluation of SafeNet^®^ and SafeNet NF^®^ LLIN

#### Test items

The test items included the polyester SafeNet^®^ and the SafeNet NF^®^ LLINs coated with alphacypermethrin; the technical grades of Piperonyl butoxide (PBO) and alphacypermethrin (ACM). The difference between the two candidate nets was the physical characteristics, with SafeNet NF^®^ having wider mesh, lower grams per square meter (GSM), higher denier, lower bursting strength, Table 1.

**Table 1:** Characteristics for study LLINs

Test item	Active ingredient	Denier	GSM	Batch no	Test facility codes
SafeNet®	200 mg/m^2^ ACM	100 denier	40 ± 10%	NTG180702.1 WT.07.18.02 (7 nets) xxx20181020-1 (7 nets) yyy20181020-2 (7 nets)	749B–762B 771B–772B 785B 788B–791B
SafeNet NF®	200 mg/m^2^ ACM	100 denier	36 ± 10%	456–20181020 (8 nets) 123–20181020 (7 nets) 789–20181020 (6 nets)	735B–748B 792B–798B
Interceptor®	200 mg/m^2^ ACM	100 denier	40 ± 10%	4934415632 (21 nets)	721B–734B 779B–784B 773B
Safi Net	N/A	Not indicated	Not indicated	No batch numbers	763B–770B 774B–778B 786B and 787B

*N/A* not applicable

The working solutions of PBO (batch number 8615500) and alphacypermethrin (ACM, batch numbers 5823400 and 8592500) were prepared from technical grade received from a commercial supplier (Chem Service Inc. West Chester PA, USA). The purity of the first sample (batch 5823400) was reported as 98.4% ± 0.5%, and the second sample (batch 8592500) as 99.5% ± 0.5%, by an ISO-certified testing facility. The purity of technical grade was reported as 98.3% ± 0.5% as indicated on the certificate of analysis. The certificates of analysis were considered sufficient for verification of integrity and quality of the technical grade insecticides.

#### Preparation of bottle bioassay working solutions

Four bottles of ACM at 12.5 µg/mL were prepared for testing in a single test and four bottles of ACM at 60 µg/mL for a separate assay test. The bottles were coated evenly following the Centers for Disease Control and Prevention (CDC) Bottle Bioassay guideline [19]. Four additional Wheaton bottles were coated with 1 mL acetone only; these were used as the negative controls. The PBO bottles were prepared in the same way, from which a dilution in acetone to 25 µg/mL was prepared. One mL of this dilution was used to coat each of 3 Wheaton bottles. All stock and working solutions were used within 24 h of preparation. Stock solutions were diluted immediately to create the working solutions, which were immediately used to coat the bottles. Likewise, once treated the bottles were used within 5 days.

#### Characterization of test systems

Bottle bioassays, biometric tests, and molecular assays were conducted to characterize mosquitoes that were used for the experimental hut trial and laboratory bioassays. Laboratory studies requires tests to confirms *Anopheles gambiae* Kisumu is susceptible, and to confirm the pyrethroid resistance in the wild-free flying *An. arabiensis*.

The wild population of *An. arabiensis* in Lower Moshi were caught at the larvae stage and reared to adults for use in bottle bioassays. In total, 300 female unfed 2–5 days old adult mosquitoes were tested against alphacypermethrin with the 1 × and 5 × diagnostic dose bottle bioassays as per CDC guidelines [19], and 150 in the PBO pre-exposure bottle bioassay (including 50 for the controls).

Test systems for cone bioassays: *An. gambiae *sensu stricto (*s.s.)* Kisumu, a fully-susceptible strain was used. Unfed 2–5 days old adult females from the insectary were characterized in terms of body weight, wing length, resistance status (phenotypic and genotypic), and species identification as outcome measures during the experimental phase of the study.

A total of 88 *An. gambiae* Kisumu mosquitoes were tested for species identification and knock down resistance *(kdr) E* genotype using quantitative real-time Polymerase Chain Reaction (qPCR) technique. Deoxyribonucleic acid (DNA) was extracted from the *Anopheles* spp using the modified chelex extraction method by Walsh [20]. Identification of the members of the *An. gambiae *sensu lato (*s.l.)* species complex was performed using the Taqman 3-plex assay of Bass et al. [21]. Detection of *kdr* mutations was performed using the Taqman assay method [22]. A separate sample of 100 *An. gambiae* Kisumu was used for the biometric characterization of the colony following the modified methods by Yeap [23] and Nasci [24].

#### Washing and preparation of LLINs for field trial

Whole nets were washed following WHOPES guidelines [25]. In brief, each net was washed in Savon de Marseilles soap solution for 10 min: 3 min stirring, 4 min soaking, then another 3 min stirring. This was followed by 2 rinse cycles of the same duration with tap water only. The mean water pH was 6 for all washes. The mean water hardness was 50.4 parts per million (ppm) and always within the WHOPES limit of ≤ 89 ppm. Seven nets of each treatment (Table 1) were washed for use in the hut study, and one additional net was washed and retained/ ‘held back’ (HB), which is not used in the hut study (Table 2).

**Table 2:** The experimental hut trial treatment arms

Treatment arm	Washes	Used in hut trial	Nets held back
Reference item			
Interceptor® ^®^ LLIN	× 0	721B–727B	727B
Interceptor® ® LLIN	× 20	728B–734B	731B
Test items			
SafeNet NF®	× 0	735B–741B	736B
SafeNet NF®	× 20	742B–748B	747B
SafeNet® LLIN	× 0	749B–755B	753B
SafeNet® LLIN	× 20	756B–762B	757B
Negative control			
Safi net	× 20	763B–769B	768B

All nets used in the experimental hut study had 30 holes (4 × 4 cm) cut in them to simulate the conditions of a torn used net. This is a modified method by the TF to increase the number of mosquitoes that penetrate the control net to take blood meal, as previous studies at this site indicated that *An. arabiensis* exhibits very low (< 35%) net penetration and blood feeding rate [26, 27].

Five pieces 30 × 30 cm were cut from nets held back (one net for each treatment arm) before and after they were washed, 10 pieces in total per net (HB0–HB9). At the conclusion of the hut trial, hut used (HU) nets were returned to the Test Facility and five pieces (HU1–HU5) were cut from one net randomly chosen from each treatment arm. In all instances, pieces were cut from pre-defined positions on the nets. Pieces were wrapped in aluminium foil and kept in a fridge in test room 2 at 5 ± 3 °C until needed for assays.

#### Running the experimental hut trial

The experimental hut study was conducted from June 2019 to August 2019 at the Pasua Field Station in seven huts.

In brief, treatments and baits (cows used to attract mosquitoes into experimental huts) were randomized using a 7 × 7 Latin square design (https://www.dcode.fr/latin-square). The treatments were rotated weekly, with a daily rotation of replicate nets of the same treatments, while the cows were rotated daily. Cows were kept inside the experimental huts for 12 h (from 6:30 pm to 6:30 am), and a collection of mosquitoes was conducted in the morning. Mosquitoes were collected daily from the window exit traps, verandas, room, and inside the net from each of the 7 huts. Both live and dead mosquitoes were collected in separate cups for the live, blood-fed, unfed, dead and their point of location recorded. All mosquitoes caught alive were kept in paper cups provided with glucose solution 10%. Mortality was scored 24 h later. On every 8th day before changing treatments, the huts were cleaned, and left open for ventilation to prevent carry over effects between treatments.

#### WHO cone bioassays

Cone bioassays for pieces cut from hut trials used and unused net were conducted in accordance with the standard WHOPES guidelines [25] using the 2–5 days old susceptible *An. gambiae* Kisumu. In brief, 2 replicates for each of the 5 pieces per net were run with 5 mosquitoes per cone, making a total of 50 mosquitoes for each net representing each treatment arm. The negative control pieces were tested alongside the test items and reference items. Any replicates for which control mortality exceeded 10% at 24 h were not analysed, and further replicates were carried out to replace those that were excluded. For all bioassays, there was a minimum of one hour holding period pre-exposure and a 3-min exposure time. After exposure, mosquitoes were released into holding cups and provided with 10% glucose solution, 60 min knockdown, and 24 h mortality were recorded.

#### Bottle bioassays

The CDC bottle bioassay guideline [19] was followed for both (a) the ACM-only bioassays and (b) the PBO pre-exposure bottle bioassays. In brief, in the ACM-only assays mosquitoes were exposed directly to ACM 1 × or 5 × (4 bottle of each concentration, 25 ± 3 mosquitoes/bottle). In the PBO pre-exposure assays mosquitoes were exposed to acetone and PBO (25 µg/mL) for 1 h, then held in cages for 1 h before exposure to ACM 1× (4 bottles, 25 ± 3 mosquitoes/bottle) and acetone (2 bottles, 25 ± 3 mosquitoes/bottle).

During the 30 min exposure to ACM or acetone, knockdown was recorded at 0 min and then every 5 min until 30 min. After 30 min, mosquitoes were removed from the bottles, transferred back into the holding cups, and provided with glucose solution 10%. At 24 h post-exposure mosquitoes were scored as dead or alive.

#### Data management and analysis

The number of mosquitoes per bottle and cones was within the acceptable range of 25 ± 3 and 5 ± 1 mosquito respectively. The control mortality for cones and bottle bioassays were all less than 10%, therefore, mortality in the insecticide treatment groups was not adjusted.

The 95% confidence intervals for proportionate data were calculated from the proportion of observations of interest (number knockdown/number dead), the total observed, assuming α = 0.05, using the following formula:

$$confidence\,interval=\sqrt{\left(\frac{proportion\times (1-proportion)}{total\,observed}\right)} \times 1.96$$

Double entry, comparison checks, and accuracy checks on all the datasets were carried out in MS Access 2016. All datasets were transferred into Stata I/C v11.0 (Stata Corp LLC, Texas USA) statistical software using Stat Transfer v8.0. Graphs were created in MS Excel 2016.

The data for the free-flying mosquito collections in the hut trial were analysed using logistic regression for grouped data with odds ratio output, adjusting for effects of hut and sleeper. Statistics were run using Stata/IC version 11.1 (Stata Corp LP College Station TX77845, USA). The main outcomes from the experimental huts were: deterrence, blood-feeding inhibition, and mortality. The 2-sample *t*-test was used to compare the mortality data between candidate net and reference nets at both 0 and 20 times washes for the pieces derived from hut used nets (see Additional file 1).

## Results

### GLP procedures

#### Study risk assessment and mitigation

Activities with potential hazards identified were; net washing, cone and bottle bioassays, and mosquito collection during hut trial. Likelihood and severity were scored as 2 and 1 respectively, and risk level were assigned for each activity Table 3.

**Table 3:** Risk assessment for the study

Activity	Hazard	Existing controls	Risk level
Net cutting and labelling	Contact with net treated with alphacypermethrin	Laboratory coat, gloves, dust masks	Low
Net washing	Contact with net treated with alphacypermethrin, and water from washed nets	Laboratory coat, gloves,	Low
Cone bioassays	Contact with net treated with alphacypermethrin	Laboratory coat, gloves,	Low
Working solution preparation and bottle coating	Contact with technical grade alphacypermethrin, inhaling fumes from alphacypermethrin	Laboratory coat, gloves, half mask respirators and laboratory shoes	Low
Bottle bioassays	Contact with bottles coated with alphacypermethrin	Laboratory coat, gloves and laboratory shoes	Low
Hut trial	Contact with net treated with alphacypermethrin	Laboratory coat, gloves, gum boots	Low

Persons potentially at risk were identified. Gloves, gumboot, overalls were recommended as appropriate personal protective equipment for whole net washing while lab shoes, laboratory coats, and gloves were recommended for use during laboratory bioassays. The overall risk score for the three study activities assessed was 1, which implies low risk based on a 0–5 scale.

#### Critical phase auditing

The 10th wash of the whole nets and all previous washing from gathered records complied with SOPs and study plan. Furthermore, the conduct of cone assays and the rotation of treatments and the collection of mosquitoes in the hut trial, and scoring for primary outcome measures during the hut trial were found to conform with SOPs and study plan. Equivalently, the audit of the experimental determination of alpha-cypermethrin in LLINs was found to comply with SOPs and study plan at the test site. Both TF and TS confirmed that staff involved in the study were trained and competent, relevant SOPs, study plan, test item and test system record sheets were available at the study area. Equipment were also found to be timely maintained and calibrated.

#### Data management, software validation, and accuracy checks

All data did not deviate post cleaning at the time of analysis in all the software non-errored computer systems post validation.

#### Study compliance to GLP

The study was audited internally and externally by SANAS as part of the annual audit inspection of the Test Facility and no serious study-related non-conformances were found. This study complied with OECD-GLP standards.

#### Experimental hut study

One thousand and forty-seven (1047) free-flying *An. arabiensis* were collected from the experimental huts on 49 nights from June to August 2019. Mosquito entry rates in the huts with unwashed Interceptor^®^ LLIN, 20 W washed Interceptor^®^ 20 W LLIN, SafeNet NF and 20 W Safenet LLIN were reduced compared to negative control hut (untreated Safi net) at 46.4%, 32.1%, 12.9% and 3.5% respectively. Deterrence was not observed for the other arms of the trial (Table 2). Similar exiting rates were found for all the treatment arms and no significant induced exiting was detectable because the exiting rate in the control hut was very high,—89.4% which could be accounted by the vector’s natural early-exiting behaviour. The early-exit behaviour has been reported for this vector at this site [26, 27] and elsewhere [28]. There was significant blood-feeding inhibition in the Interceptor^®^ LLIN treatment arms (0 W and 20 W) but not in other arms. Blood feeding inhibition was 42.5% for Interceptor^®^ LLIN 0 W and 35.8% for Interceptor^®^ LLIN 20 W, though after adjustment for the effects of hut and sleeper the latter was found to be not significantly different. However, in the control hut, the proportion of blood-fed female mosquitoes was only 30.6% compared to the total number captured. All insecticide treatments, Interceptor^®^ (0 W), Interceptor^®^ (20 W), SafeNet NF^®^ (0 W), SafeNet NF^®^ (20 W), SafeNet LLIN (0 W) and SafeNet (20 W), induced significantly higher mortality compared to the negative control at 25.50% (95% CI 16.5–34.5), 23.20% (95% CI 15.4–31.0), 27.97%(95% CI 21.4–34.6), 15.22%(95% CI 9.4–21.0), 16.30% (95% CI 11.0–21.6) and 14.90% (95% CI 9.4–20.3) respectively (Table 2). For all treatment arms mortality decreased with washing, although this reduction was only significantly different for SafeNet NF^®^.

In a comparison of the six insecticidal treatments with each other, the performance of SafeNet NF^®^ was not statistically different from the reference arm (0 W and 20 W Interceptor^®^ LLIN). SafeNet^®^ LLIN 0 W had an equivalent performance to Interceptor® LLIN 0 W and 20 W (Table 4).

**Table 4:** Deterrence, exophily, blood feeding and mortality in the 7 treatment groups

Treatment arms							
	Control	Interceptor^®^ 0 W	Interceptor^®^ 20 W	SafeNet NF^®^ 0 W	SafeNet NF^®^ 20 W	SafeNet^®^ LLIN 0 W	SafeNet^®^ LLIN 20 W
Deterrency and exophily							
No. females caught	**170**	**91**	**112**	**177**	**148**	**185**	**164**
Deterrency (%)	–	46.5	34.1	−4.1	12.9	−8.8	3.5
Room	**11**	**5**	**9**	**22**	**19**	**22**	**17**
Verandah + window	**152**	**86**	**101**	**149**	**125**	**163**	**143**
Net	**7**	**0**	**2**	**6**	**4**	**0**	**4**
Inside net (%)	4.1	0.0	1.8	3.4	2.7	0.0	2.4
Exophily (%)	89.4	94.5	90.2	84.2	84.5	88.1	87.2
95% CI	84.8–94.0	89.8–99.2	84.7–95.7	78.8–89.6	78.6–90.3	83.4–92.8	82.1–92.3
*P*		0.17	0.84	0.15	0.19	0.70	0.53
Induced exophily (%)	–	NS	NS	NS	NS	NS	NS
Blood feeding							
Blood fed	**52**	**16**	**22**	**55**	**39**	**42**	**53**
Blood fed (%)	*30.6*^*a*^	*17.6*^*b*^	*19.6*^*ab*^	*31.1*^*a*^	*26.4*^*a*^	*22.7*^*ab*^	*32.3*^*a*^
95% CI	23.7–37.5	9.8–25.4	12.3–27.0	24.3–37.9	19.3–33.5	16.7–28.7	25.2–39.5
*P*		0.02	0.04	0.92	0.40	0.1	0.7
Blood feed inhibition (%)	–	42.5	35.8	NS	NS	NS	NS
Mortality							
Overall mortality	**2**	**24**	**27**	**51**	**24**	**32**	**26**
Overall mortality (%)	*1.2*^*a*^	*26.4*^* cd*^	*24.1*^* cd*^	*28.8*^*d*^	*16.2*^*bc*^	*17.3*^*bc*^	*15.9*^*b*^
95% CI	0–2.8	17.3–35.4	16.2–32.0	22.1–35.5	10.3–22.2	11.9–22.8	10.3–21.4
*P*		0.00	0.00	0.00	0.00	0.00	0.00
Corrected for control (%)	–	25.50	23.20	27.97	15.22	16.3	14.9
95% CI	–	16.5–34.5	15.4–31.0	21.4–34.6	9.4–21.0	11.0–21.6	9.4–20.3
Mortality of unfeds	**2**	**21**	**23**	**43**	**20**	**26**	**21**
Ratio of dead/total dead (%)	100.0	87.5	85.2	84.3	83.3	81.3	80.8
Unfed dead/total (%)	1.2	23.1	20.5	24.3	13.5	14.1	12.8
*P*		0.00	0.00	0.00	0.0	0.00	0.00
Corrected for control (%)	–	22.2	19.59	23.4	12.5	13.0	11.8
Immediate mortality	**0**	**9**	**8**	**18**	**7**	**11**	**11**
Ratio imm/total mortality (%)	0.0	37.5	29.6	35.3	29.2	34.4	42.3
Imm mortality/total (%)	0.0	9.9	7.1	10.2	4.7	5.9	6.7
*P*		0.00	0.00	0.00	0.0	0.0	0.0
Corrected for control (%)	–	9.9	7.1	10.2	4.7	6.0	6.7

Main outcomes are given in Italics

*NS*  not significant

Results between LLINs which share the same super scripts a, ab,c, cd, d are not significant different

#### Cone assays for net pieces

**Fig. 4:**
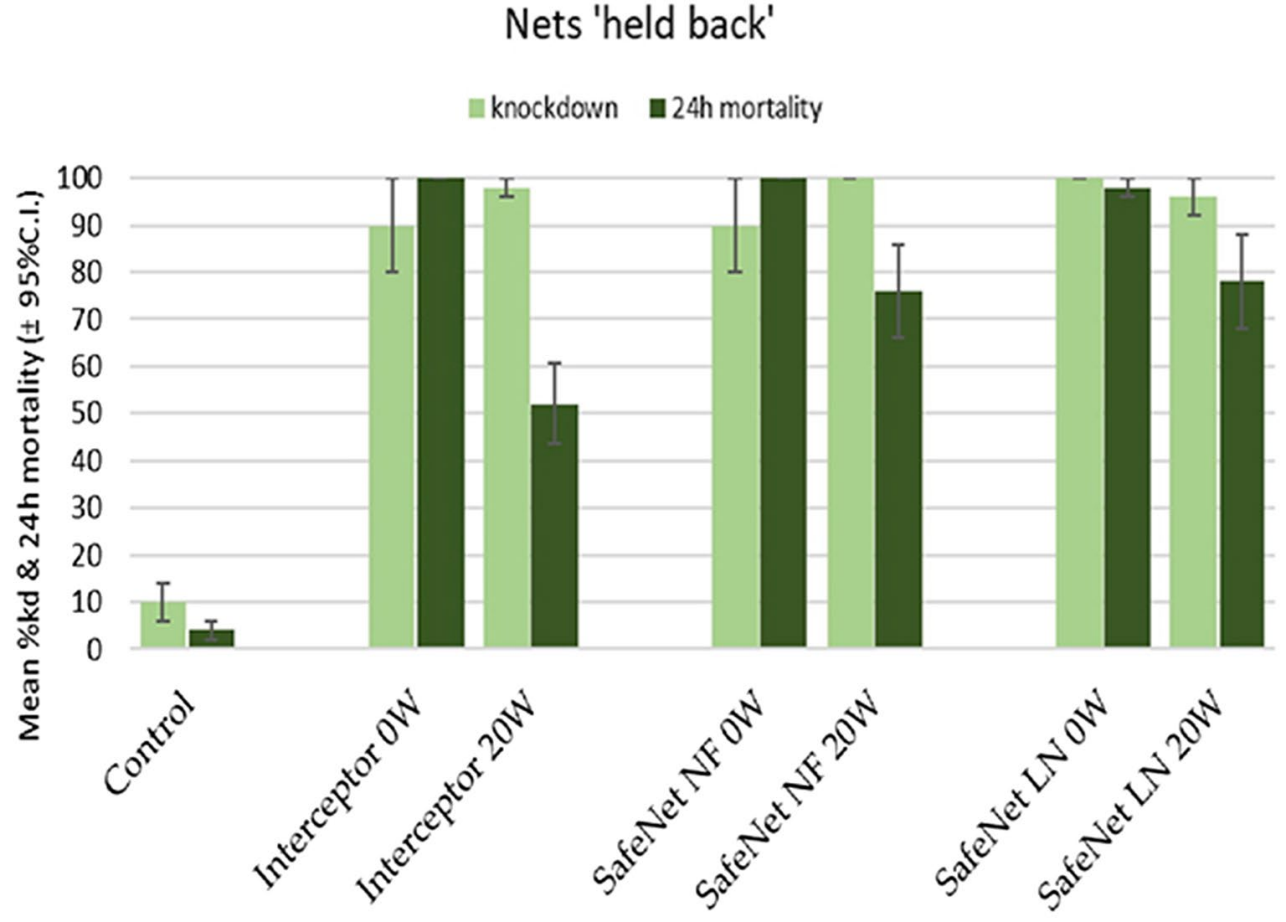
The mean 60 min *knockdown* and 24 h mortality for the *An. gambiae* Kisumu strain, after 3-min exposure in cone assays against pieces cut from held back nets

The mean knockdown and/or 24 h mortality induced by net pieces, cut from all insecticide-treated nets ‘held back’ and ‘unused’ from the hut study passed the standard WHOPES cut-off criteria (either 24 h mortality ≥ 80% or knockdown ≥ 95% or both), at either unwashed and/or washed 20 times, where Interceptor^®^ unwashed (mortality 100%), Interceptor^®^ 20× washed (Kd 98%), SafeNet NF^®^ unwashed (mortality 100%), SafeNet NF^®^ 20× washed (Kd 100%), SafeNet^®^ LLIN unwashed (Kd 100%, mortality 98%), SafeNet^®^ LLIN 20× washed (Kd 96%) (Fig. 4). SafeNet^®^ LLIN 20 W and SafeNet NF^®^ 20 W induced higher mortality than the reference net at 20 W (Table 5).

**Table 5:** The mean knockdown and 24 h mortality induced by net pieces, cut from nets ‘held back’

Nets	Number washed	Mean	95% Conf. Interval		p-value
			Lower	Upper	
Interceptor®	0	76	59.76	92.24	0.0036
SafeNet LLIN®		100	100	100	
Interceptor ®	20	84	74.95	93.05	0.0225
SafeNet LLIN®		96	89.97	102.03	
Interceptor®	0	76	59.76	92.24	0.0036
SafeNet NF®		100	100	100	
Interceptor®	20	84	74.95	93.05	0.3823
SafeNet NF®		90	77.84	102.16	

The efficacy of the 20 W pieces for all treatments appeared lower than their unwashed counterparts. Nevertheless, these pieces still induced very high knockdown rates (all above 95%). In total, 798 females unfed 2–5-day old *An. gambiae* Kisumu were exposed in the cone bioassays: 398 for pieces from used net (‘HU’ pieces) and 400 for unused net (‘HB’ pieces).

**Fig. 5:**
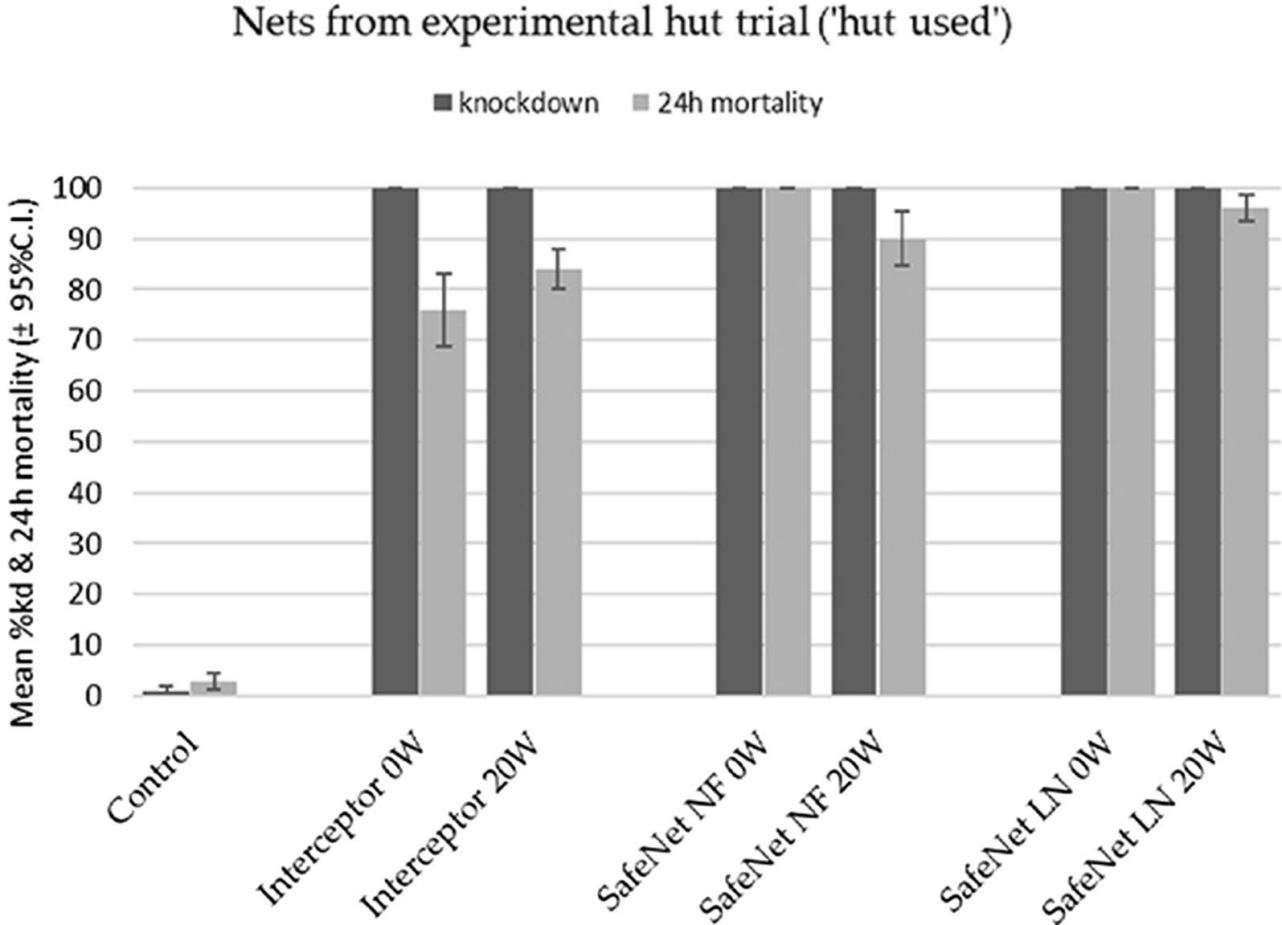
Mean percentage 60 min knockdown and mean 24 h percentage mortality for *An. gambiae* s.s. Kisumu strain, after 3-min exposure in cone assays against pieces cut from hut-used nets

Mean knockdown and mortality induced by net pieces cut from randomly-selected insecticide-treated nets used in the hut trial also passed the standard WHOPES cut-off criteria (mortality ≥ 80% and/or knockdown ≥ 95%). The 0 W and 20 W hut-used SafeNet^®^ LLIN and SafeNet NF^®^ passed both the knockdown and mortality cut-offs. The Interceptor^®^ LLIN 0 W passed on knockdown but failed mortality (Fig. 5, Table 6).

**Table 6:** The mean knockdown and 24 h mortality by hut-used and held back LLINs

Net type	% Mortality		
	No. of washes	Held back	Hut USED
Interceptor® LLIN	Unwashed	100.0	75.3
	20 × washed	50.0	83.5
SafeNet NF®	Unwashed	100.0	100.0
	20 × washed	75.0	89.7
SafeNet® LLIN	Unwashed	97.9	100.0
	20 × washed	77.1	95.9

#### Bottle bioassays

**Fig. 6:**
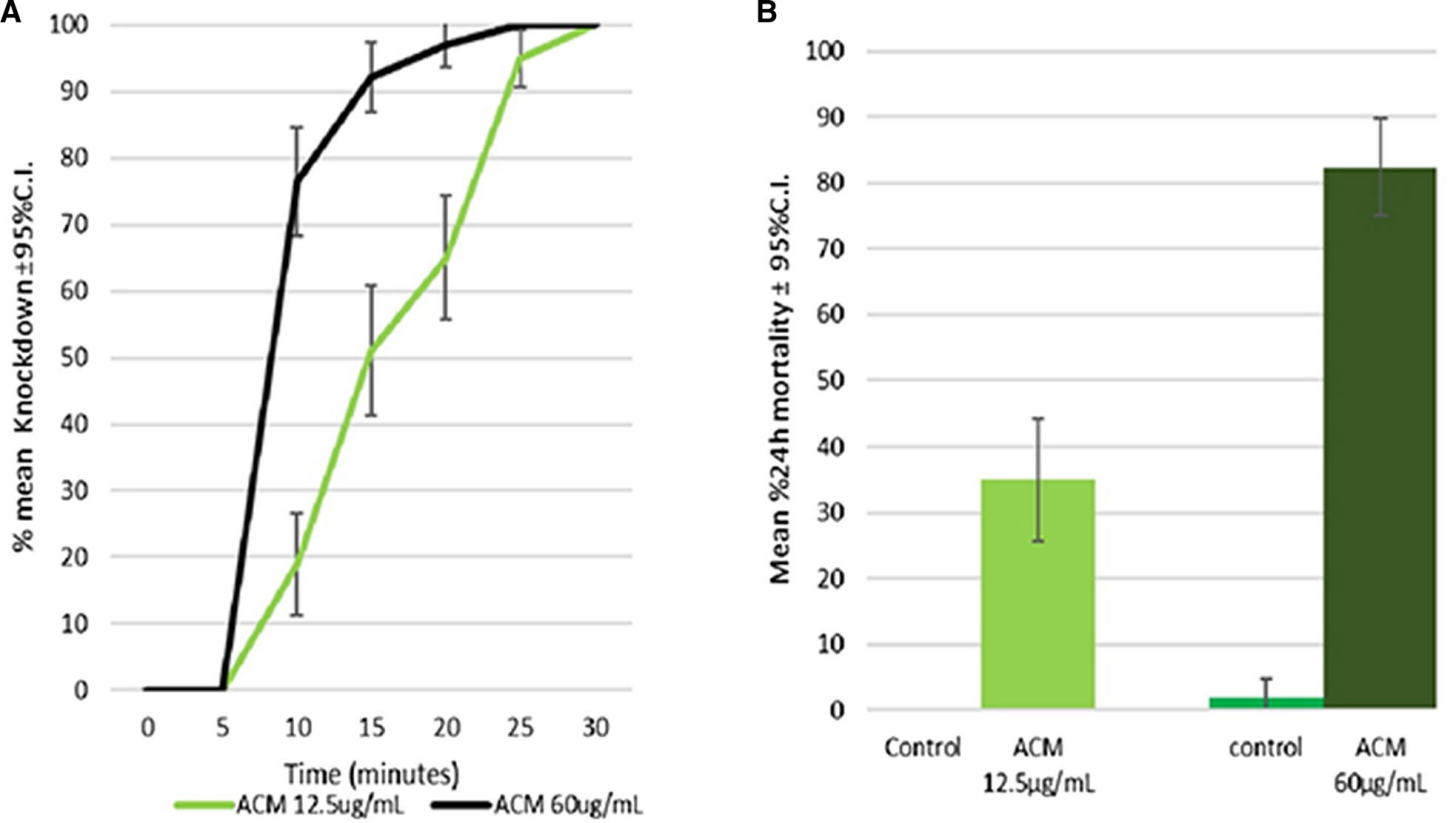
**a** Mean percentage knockdown **b** mean percentage mortality at 24 h, for *An. arabiensis* after 30 min exposure to ACM at 12.5 µg/mL (1 × diagnostic dose) or 60 µg/mL (5 × diagnostic dose)

All *An. arabiensis* exposed to 12.5 µg/mL ACM were knocked down within 30 min, but then showed recovery post-exposure: average mortality at 24 h was 35.0%. This suggests the pyrethroid-resistance status of the wild caught *An. arabiensis* as per WHO criteria [29]. Resistance was exhibited by *An. arabiensis* in separate test with exposure to 5 × diagnostic dose (60 µg/mL ACM). All mosquitoes (102) were knocked down within 25 min (Fig. 6a) but only 82.4% were dead at 24 h (Fig. 6b.). This reflects metabolic resistance which was previously known about this test system [17] and was reconfirmed in subsequent PBO bottle bioassays and molecular lab assays during this study.

For the *An. gambiae* Kisumu strain; 24 h post-exposure mortality after a 30-min exposure to ACM at 12.5 µg/mL was 100% in all three assays. This confirms the pyrethroid-susceptibility status of the test system. Susceptibility is demonstrated by the mortality of ≥ 98%. Post-exposure mortality after a one-hour exposure to Mero®-acetone (negative control) vehicle only was always < 20%—the maximum value was 17.4%.

#### Synergist bottle bioassays

**Fig. 7:**
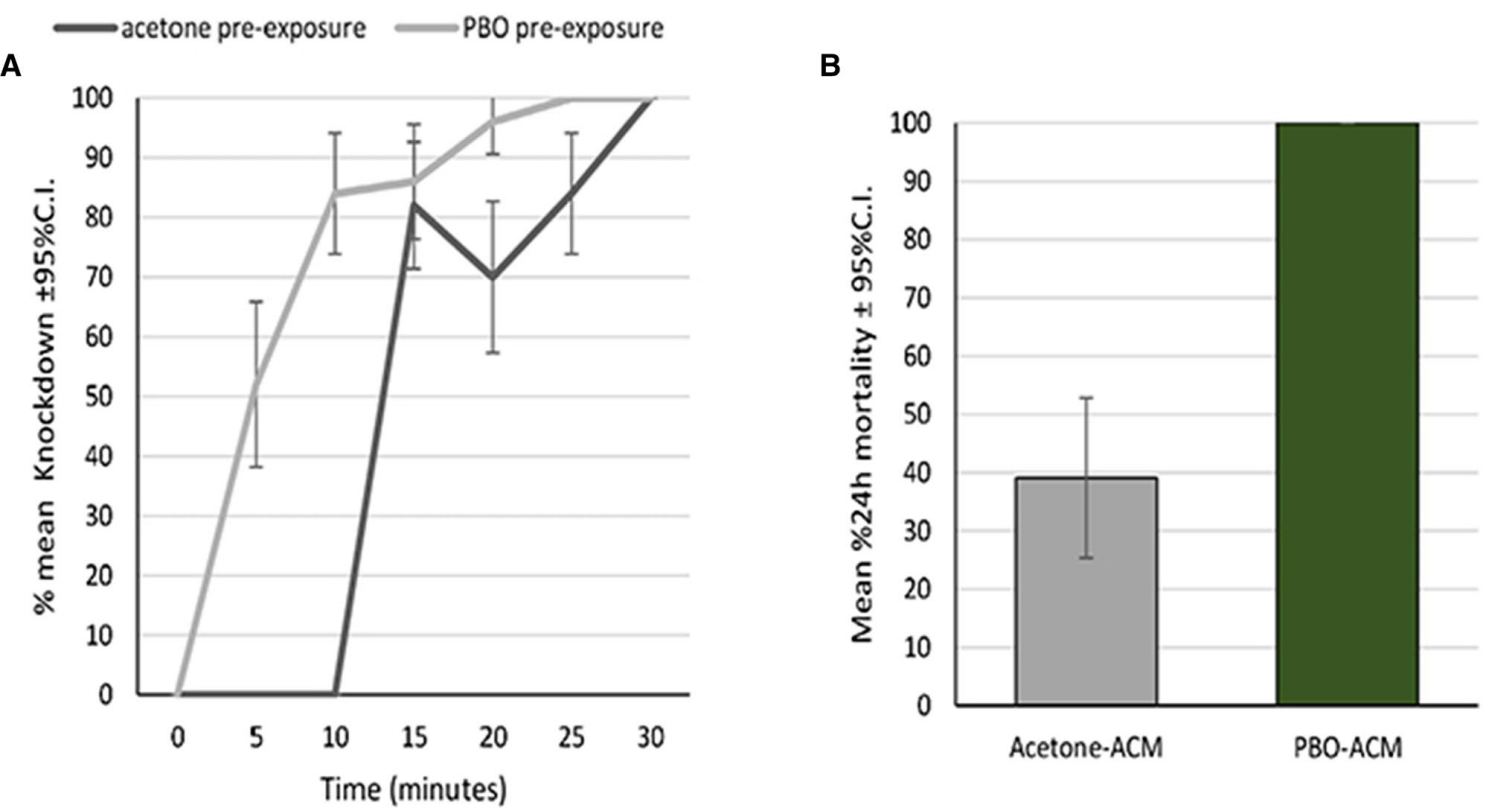
**a** Mean percentage knockdown and **b** control adjusted-mean percentage mortality at 24 h, for *An. arabiensis* after 30-min exposure to ACM at 12.5 µg/mL, following a 1 h pre-exposure to acetone or PBO

The results for the mortality for the *An. arabiensis* against 12.5 µg/mL ACM without synergist pre-exposure and with synergist pre-exposure to PBO was 35% (with control adjusted mortality) and 100% respectively (Fig. 7). This indicates the presence of metabolic resistance mechanism(s), involving overexpression of coded P450 genes.

#### Molecular characterisation of the test system

Results indicate that 100% of mosquitoes used for the cone bioassays were *An. gambiae s.s*., and were all homozygous susceptible, *SSe,* meaning no mosquito was homozygous or heterozygous for the *kdr* L1014S gene, Table 7. This further indicates that there was no evidence of strain contamination and that the strain was confirmed suitable for cone bioassays*.* Eighty-eight of the wild-caught mosquitoes were assayed in the same way: 100% were shown to be *An. arabiensis* and no individuals were homozygous or heterozygous for the *kdr* L1014S gene, which means all samples were *SSe* status.

**Table 7:** Results for the test systems’ species and *Kdr* identification

Test system	Species			*Kdr*		
	*An. gambiae s.s*	*An. arabiensis*	Other	*RRe*	*RSe*	*SSe*
*An. gambiae s.s.* Kisumu	88	0	0	0	0	88
*An. arabiensis* (wild caught)	0	88	0	0	0	88

#### Biometric characterisation of the test system

**Fig. 8:**
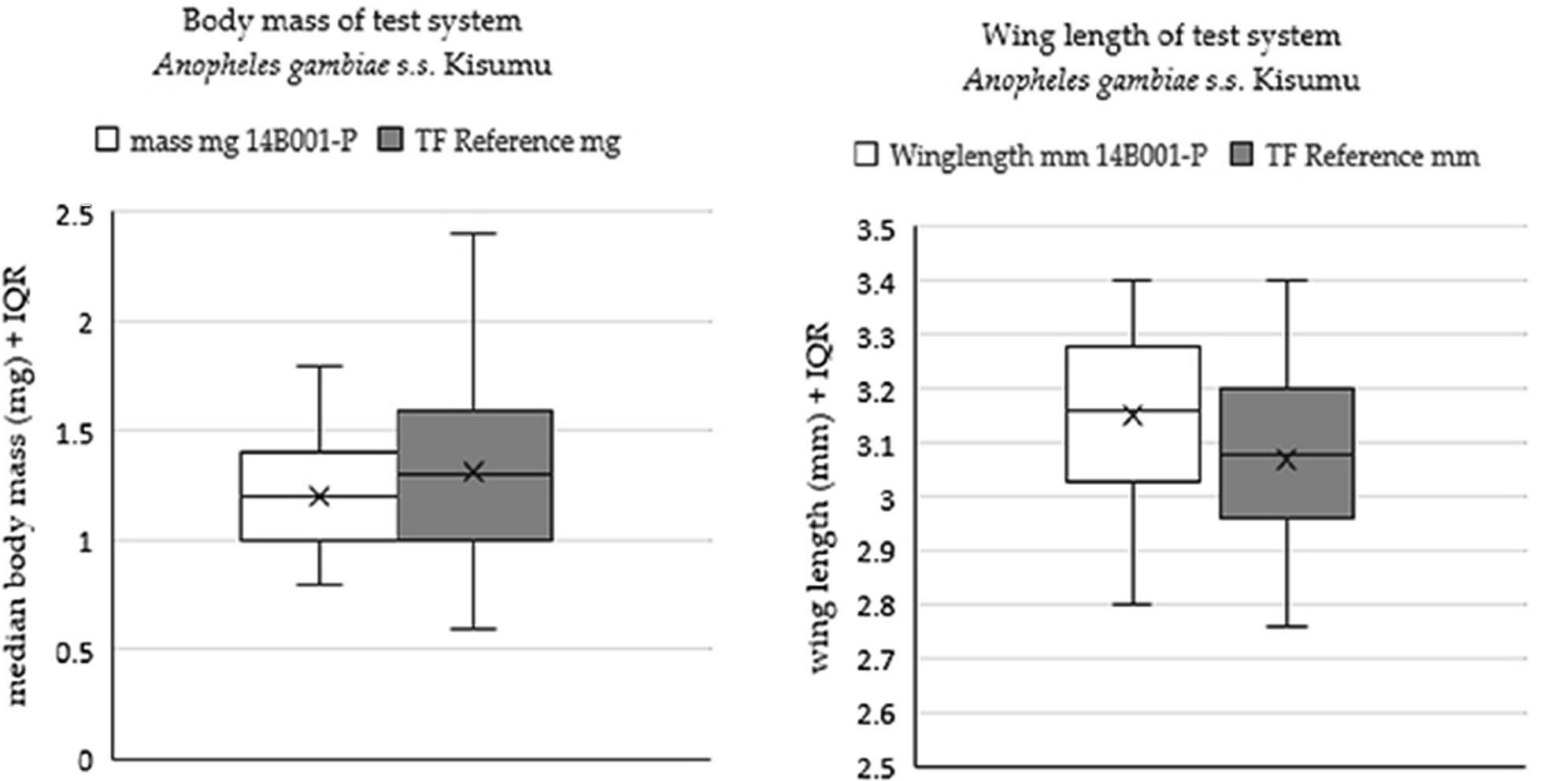
Box and whisker plots for (from left to right) mosquito body mass and wing length. Means are indicated by X. TF = Test Facility. IQR = Interquartile range

The mean ± 95% confidence interval for wing length for 50 *An. gambiae* Kisumu was 3.15 mm (3.11–3.19 mm). The mean ± 95% confidence interval for body weight was 1.21 mg (1.13–1.28 mg). These means for the biometric measurements are within the 25–75% interquartile range data generated from routine biometric screening of the strain (see Fig. 8), which interprets a normal expected size and weight for mosquitoes reared at the TF based on the established SOPs. Since *An. gambiae* Kisumu data for wing length and weight were within this target biometrics, they were considered suitable for bioassays.

## Discussion

The conduct of the multi-site GLP compliant study on the evaluation of LLINs in an African context is detailed here, with special considerations to the practical aspects. The study was audited internally and externally without finding major non-conformances. It was thus concluded that the study was compliant with OECD-GLP standards. The importance of GLP standards in vector-control test facilities currently being addressed worldwide. After the KCMUCo-PAMVERC test facility achieved GLP compliance in 2017, at least two more Test Facilities that test vector control products have been granted GLP certification [30, 31], and several more are going through a similar transition [9] supported by the Innovative Vector Control Consortium or the WHO. Having multiple test facilities operating to the same international standard is crucial for the effective and reliable evaluation of novel vector control tools. Upon reflection it was found that delays caused by the multi-site nature of the study featured a critical challenge. This is mainly attributable to the lack of appropriate equipment or collaborative research centres with qualifications to do chemical analysis in-country, highlighting the need for capacity strengthening for the African research centres [32, 33]. More investments are needed to accommodate GLP-compliant HPLC capacity within African research institutions participating in vector control product evaluation. Such investment will evade high costs to abroad contracted facilities, delays, and communication-associated challenges. Recently witnessed reductions in the investment for control of malaria is further impaired by the Coronavirus disease (COVID-19) pandemic which has disproportionately affected the donor countries [34], causing a negative effect on malaria and other disease control programs [35–37]. Therefore, taking a stance to generate new LLINs at reduced manufacturer production costs could help in increasing LLIN production.

The efficacy in experimental huts of the new ACM net, SafeNet NF^®^, expressed as 24 h mortality of mosquitoes, was similar to that of the reference Interceptor® ^®^ LLIN and consistent with the results anticipated for a pyrethroid-only net used in a setting where the predominant vector species, *An. arabiensis* expresses metabolic resistance to pyrethroids but no knockdown resistance with a high exophilic behaviour. For SafeNet^®^ LLIN, the performance of the unwashed net was not significantly different from the positie control, however after 20 washes induced mortality by SafeNet^®^ LLIN was 15.9%, which was significantly lower than the positive control, which showed 24.1% mosquito mortality. The chemical analysis results did not give a conclusive answer on the active ingredient content of the net pieces used in the study and should be repeated to examine whether this reduction in efficacy is due to the active ingredient being lost after washing. As none of the insecticide treatment arms induced 30% mortality against the pyrethroid-resistant wild type mosquitoes, it is debatable whether any of the marginal statistically significant differences detected between the treatment arms are biologically or epidemiologically relevant.

Deterrence in mosquito entering the hut, relative to the control, was observed for both Interceptor® LLIN arms, but was not a major factor in the treatments with SafeNet NF and SafeNet LLIN. Similar high exiting rates were found for all the treatment arms which could be accounted by the vector’s natural early-exiting behaviour [26, 27]. There was significant blood-feeding inhibition in the Interceptor^®^ LLIN treatment arms, but not in the SafeNet NF or SafeNet LLIN arms, compared to the untreated control. However, despite the increase in number of holes per net, the proportion of blood-fed mosquitoes in the control arm was only 30.6%. Although results indicate that Interceptor^®^ LLIN induces blood feeding inhibition, low blood feeding rate in the control arm suggests other factors could be affecting mosquito blood feeding. Low blood feeding rate (< 35%) by *An. arabiensis* in this area has been reported in previous studies [26, 27].

In the cone bioassays, pieces cut from unused nets and hut-used candidate nets passed either of the performance thresholds laid down by WHO i.e. 24 h mortality ≥ 80% and knockdown ≥ 95%, against the pyrethroid-only *An. gambiae* Kisumu [25]. This was the case for both unwashed and 20 × washed pieces.

Taking laboratory and experimental hut trial results into consideration, there is no conclusive evidence to suggest a better overall performance of one net type over the others. It is reasonable to say the bio efficacy and wash resistance of both SafeNet^®^ LN and SafeNet NF^®^ are broadly comparable to the reference product (Interceptor^®^ LN), whilst acknowledging there were some inconsistencies and unforeseen deviations from expected performance in both candidate and reference nets.

## Conclusions

This study demonstrated that GLP-compliant evaluation of vector control products can be successfully carried out by African research institutions. Equivalent performance of the candidate nets with reference nets provides promise that these candidate nets can be considered to facilitate universal LLIN coverage. Caution should be taken though when using the candidate nets in areas with strong metabolic resistance. Community or equivalent trials should be done to understand the durability, acceptability, and residual efficacy of these candidate nets at the field context.

## Limitations

Results from the chemical analysis were not presented in this paper due to disagreement on methodology adopted by the TS. Results could have added more insight on the insecticide dynamics and helps to better interpret performance of candidate nets. Data from the present study were insufficient for explaining the observed blood feeding inhibition and high deterrence associated with Interceptor LLIN use compared to that of candidate nets. More studies in locations with different vector species that have different resistance mechanisms are needed to confirm the blood feeding inhibition and high deterrence effects of Interceptor LLINs.

## Supplementary Information

**Additional file 1: **Infographic.

## Data Availability

Datasets generated and analysed are presented in a summarized way in this article, full datasets will be made available from the corresponding author upon rational request.
