# Supplementary figures and images for: Implementing OECD GLP principles for the evaluation of novel vector control tools: a case study with two novel LLINs, SafeNet® and SafeNet NF®
Source: Malar J. 2022 Jun 11;21:183. doi: 10.1186/s12936-022-04208-4 (PMC9188019; doi:10.1186/s12936-022-04208-4)

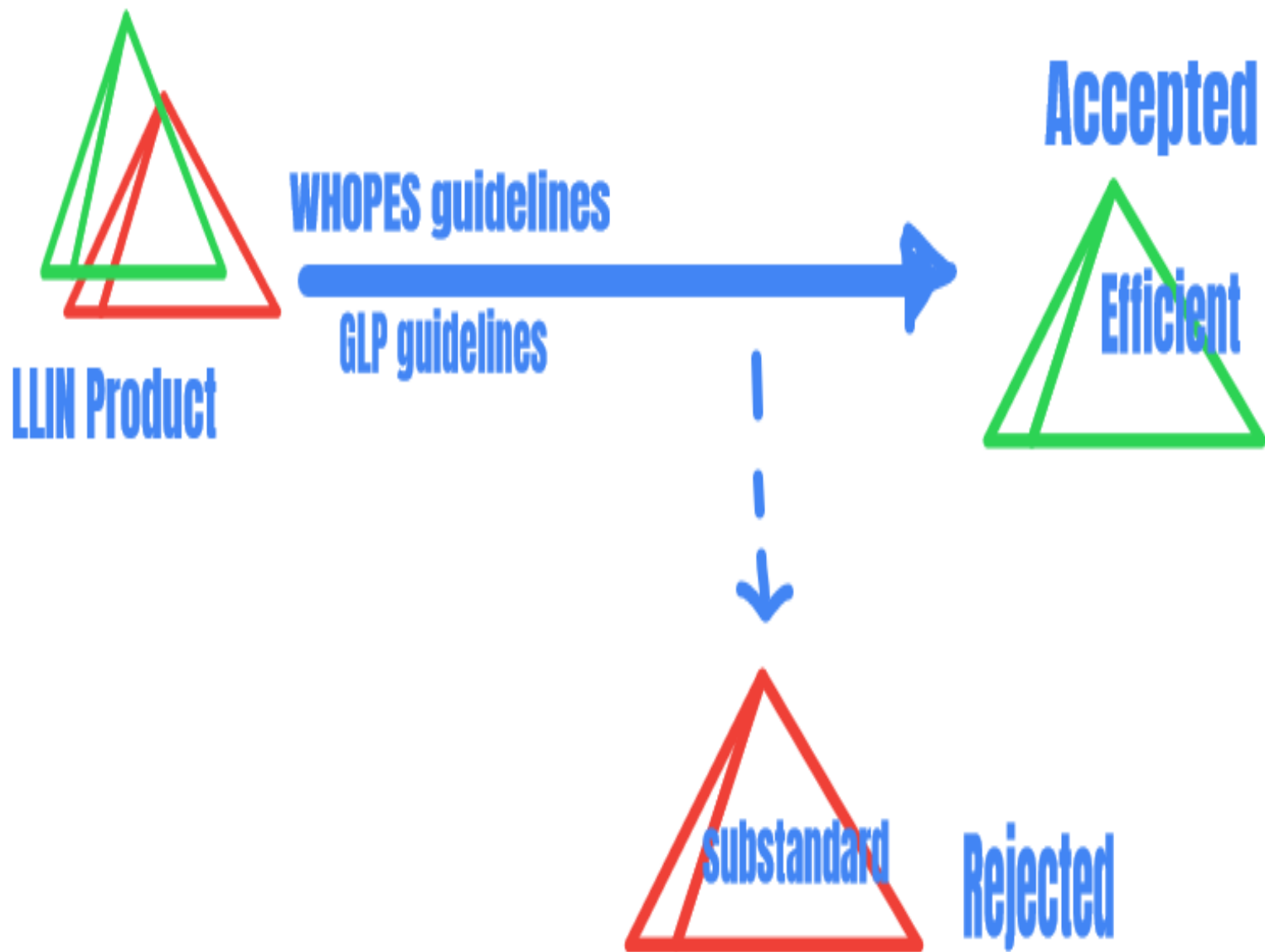

Supplement: Supplementary file 1 — Additional file 1: Infographic. [file 12936_2022_4208_MOESM1_ESM.pdf]
